# Low-Threshold-Voltage and Electrically Switchable Polarization-Selective Scattering Mode Liquid Crystal Light Shutters

**DOI:** 10.3390/polym10121354

**Published:** 2018-12-06

**Authors:** Zhe-Yung Liang, Ching-Yen Tu, Tsung-Hsun Yang, Cheng-Kai Liu, Ko-Ting Cheng

**Affiliations:** Department of Optics and Photonics, National Central University, 320 Taoyuan, Taiwan; c122388827@gmail.com (Z.-Y.L.); best841217@gmail.com (C.-Y.T.); thyang@dop.ncu.edu.tw (T.-H.Y.)

**Keywords:** liquid crystals, polarization, scattering, polymers

## Abstract

Low-threshold-voltage (V_th_) and electrically switchable, polarization-selective scattering mode light shutters (PSMLSs) using polymer-dispersed liquid crystals (PDLCs) are demonstrated in this work. The optimized weight ratio of the nematic liquid crystals (LCs) to the adopted monomer (NBA107, Norland Optics) in the low-V_th_ PDLCs based on NBA107 is 7:3, [7:3]-PDLCs_NBA107_. The properties of the low-V_th_ PDLCs_NBA107_, such as light-scattering performance, initial transmission, V_th_, and droplet size were investigated. Experiment results show that the surface anchoring (threshold-voltage) of NBA107 is weaker (lower) than or equal to that of the common NOA65. The cost is that the response time of the proposed PDLCs_NBA107_ is relatively long. A method to reduce the decay time, which can be applied to all other PDLC devices, will be elucidated. In addition to the low V_th_ of the proposed PDLCs_NBA107_, the operation voltage (~6 V_rms_) to approach the maximum transmission is relatively low in a 7 μm-thick PDLCs_NBA107_ cell. Moreover, the polarization-selective light-scattering performances of the proposed PSMLSs based on the [7:3]-PDLCs_NBA107_, mainly driven by in-plane and vertical fields, are also demonstrated.

## 1. Introduction

Optical devices based on polymer-dispersed liquid crystals (PDLCs) have been studied worldwide for several decades because of their electrically switchable light-scattering properties [1,2]. PDLCs can be applied to light shutters, 2D/3D switching, displays, holographic gratings, optical retardations, and so forth [1,2,3,4,5,6,7,8,9,10,11,12]. Polymers and liquid crystals (LCs) with a proper weight ratio are initially well-mixed with each other, and LC droplets can be formed in a continuous polymer matrix through polymerization-induced phase separation (PIPS) after treatment with UV illumination for a certain duration [1,2]. With regard to the range of the visible wavelength and without the application of an external field, PDLCs scatter unpolarized visible lights because of the random distribution of the LC directors in individual droplets. Visible lights are scattered because they encounter a refractive index mismatch between LC droplets and polymers. The size of LC droplets should be around the wavelength of visible lights, indicating that the diameters of the LC droplets are in the order of approximately 0.2–10 μm [12,13]. For common PDLCs that apply an external field [1,2], LCs (∆ε > 0) in each LC droplet are rotated to be parallel to the field direction. If the refractive index of the adopted polymers is close to the ordinary refractive index (*n*_o_) of the used LCs, then incident lights do not encounter the refractive index mismatch between LCs and polymers. Accordingly, all visible lights pass through the PDLC cell. The performances of PDLCs which are based on several popular polymers produced by Norland Products Inc. (Cranbury, NJ, USA), such as NOA81, NOA68, and NOA65, have been reported in previous studies [1,6,14,15,16,17,18]. By contrast, in this study, PDLCs based on the other polymer of Norland Products Inc., namely, NBA107, are proposed for the first time. This paper reports the full investigation of the NBA107-based PDLCs.

Several methods are reported to decrease the operation voltage in PDLCs. Hsu et al. found that the operation voltage (V_op_) and threshold voltage (V_th_) of the PDLCs doped with a suitable weight percentage of ZnO nanoparticles were reduced [18]. Chan et al. indicated that the silver-coated polystyrene microspheres doped in PDLCs construct an induced electric field to enhance the effective field in PDLCs and reduce V_op_ [19]. Additionally, Silva et al. demonstrated the method for modifying the surface anchoring on the interface between LC droplets and polymers by doping specific additives into PDLCs to reduce V_op_ [20]. Drevenšek-Olenik et al. reported LC dynamics driven by in-plane electric fields in a holographic PDLC transmission-grating cell [9]. They also reported that optical retardation contributed to PDLC cells driven by in-plane electric fields [10]. Manda et al. also proposed a method to approach the polarization-insensitive PDLC grating, driven by in-plane fields [21]. To obtain interesting optical results, we used three terminal electrodes to demonstrate the optical properties of NBA107-based PDLCs (PDLCs_NBA107_) [22,23,24,25]. The coexistent system of PDLCs and polymer-stabilized LCs are also reported [26]. Recent related studies about novel fabrication of phase-separated LC-polymer composites can also be useful references on developing PDLCs [26,27,28,29].

In this study, a low-V_th_, low-V_op_, and electrically switchable polarization-selective scattering mode light shutter (PSMLS) using PDLCs is demonstrated. The monomer used herein was NBA107, the details of which will be provided in Section 3. The properties of the PDLCs_NBA107_, such as scattering performance, V_th_, initial transmission, surface anchoring, and droplet size were investigated in detail and compared with those of the PDLCs based on the popular monomer, NOA65. The weight ratio of the selected materials (LCs: monomer = 7:3) in PDLCs_NBA107_ to approach the low V_th_ and the high contrast ratio was optimized experimentally. Experiment results show that the surface anchoring of NBA107 is weaker than or equal to that of NOA65, so the V_th_ of the PDLCs_NBA107_ is relatively low. However, the response time of the proposed PDLCs_NBA107_ is relatively long. A method to reduce the response time which can be applied to all PDLC devices will also be proposed, and the fabrication processes of the PDLCs_NBA107_ are also optimized and discussed. The polarization-selective light-scattering performances of the PSMLS based on PDLCs_NBA107_, mainly driven by in-plane and vertical fields with low V_th_, are also demonstrated. The PSMLS mainly driven by an in-plane field were found to scatter the incident lights with the polarization direction parallel to the in-plane field direction, whereas the PSMLS driven by a vertical field provided polarization-independent light-scattering performance.

## 2. Operation Mechanism of Electrically Switchable PSMLS Using PDLCs

**Figure 1:**
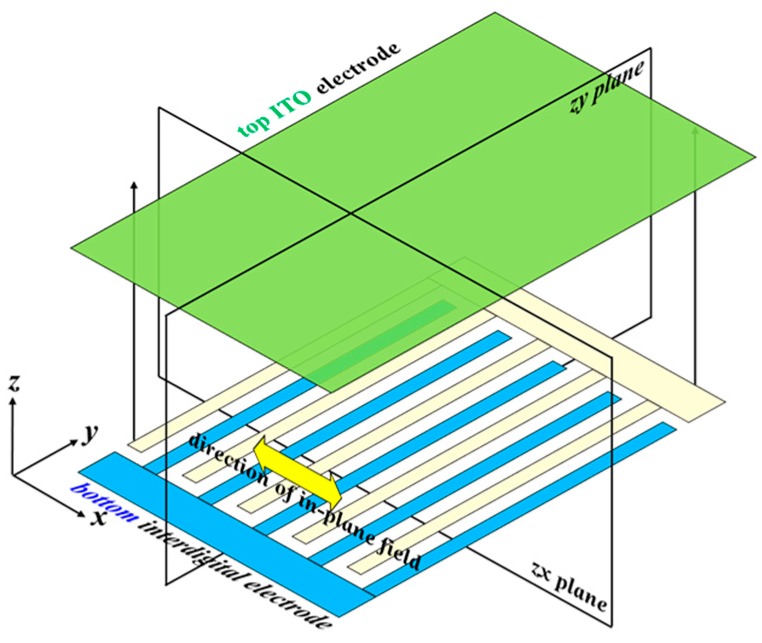
Schematic drawing of the electrode structures of the proposed polarization-selective scattering mode light shutter (PSMLS).

**Figure 2:**
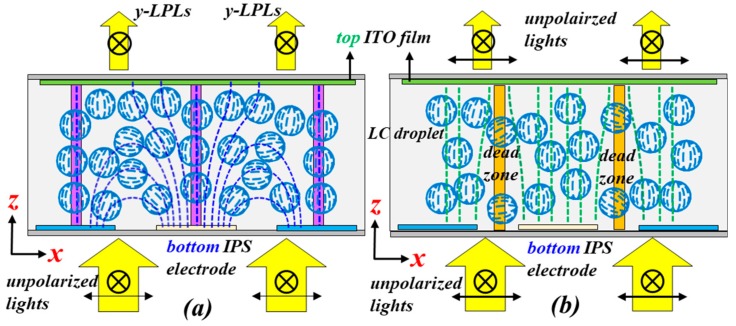
Schematic configurations of light polarization, electric field directions, and liquid crystal (LC) directors in LC droplets in the PSMLS mainly driven by (**a**) in-plane and (**b**) vertical electric fields.

Figure 1 shows the schematic diagram of the electrode structure of the proposed PSMLS. The width of the electrode stripe and the gap between two adjacent stripes are 8 and 12 μm, respectively. The bottom substrate is coated with interdigital electrode stripes to produce in-plane electric fields, whose direction is parallel to the ***x***-axis. The top substrate is coated with an indium–tin–oxide (ITO) electrode film. By properly selecting the applied electric potentials onto these three electrodes, in-plane and vertical electric fields can be easily generated and applied onto the PDLCs, as shown in Figure 2. Figure 2a presents the configurations of LC directors in LC droplets in the PSMLS, mainly driven by the two bottom interdigital electrodes. To produce in-plane fields, the electric potential on the top ITO electrode film (Figure 1) needs to be an open-circuit potential (floating), whereas the applied electric potentials on the blue and light-yellow interdigital electrode stripes (Figure 1) should be different to produce the required in-plane external fields with suitable voltages. Undesirable vertical fields should also be considered.

With regard to the case shown in Figure 2a, unpolarized lights can be divided into two orthogonal, linearly polarized lights, including the polarization directions parallel to the ±***y***- and ±***x***-axes, denoted by ***y***-LPLs and ***x***-LPLs, respectively. When suitable electric potentials are applied onto the blue and light-yellow interdigital electrode stripes (Figure 2a), in addition to the refractive index of the polymer, n_p_, most of the components of ***x***-LPLs encounter various effective refractive indices of LCs (*n*_eff_), while the components of ***y***-LPLs encounter the n_o_. This is because most of the effective electric fields between two adjacent interdigital electrodes are not parallel to the ±***x***-axis. Thus, most of the LCs in the LC droplets rotate in the ***xz***-plane with their director projection parallel to ±***x***-axis. In accordance with the basic requirement of PDLCs, the refractive index of the polymer should be close to the *n*_o_ of the adopted LCs [1]. Accordingly, under this condition, incident lights with polarization directions parallel to the effective in-plane electric field direction (***x***-LPLs) in Figure 2a are scattered because they encounter the largest refractive index mismatch between LCs and polymers [(*n*_eff_-*n*_p_) or (*n*_e_-*n*_p_)] in the PSMLS, whereas the incident lights with polarization directions perpendicular to the effective in-plane electric field direction (***y***-LPLs) can pass through the PSMLS because of the absence of a refractive index mismatch, due to the equal n_p_ and n_o_ [1,2]. However, in practical application, a small number of LCs in LC droplets close to the purple zones (Figure 2a), which are the central regions of each electrode stripe, rotate to be perpendicular to the substrates via the effective vertical fields (floating) [24,25]. Accordingly, the transmittance of either ***x***-LPLs or ***y***-LPLs is increased. As to the case with the application of the vertical electric field, Figure 2b shows the detailed configurations of the proposed PSMLS applied with a vertical electric field provided by the bottom interdigital electrode stripes and top ITO electrode film. The electric potentials applied onto the blue and light-yellow interdigital electrode stripes (Figure 1) are the same, but different from that applied to the top ITO electrode film. Therefore, the direction of the applied electric fields in most regions is perpendicular to the substrates, i.e., vertical electric fields, but the effective electric fields close to the edges of the electrode stripes are not exactly perpendicular to the substrates. Thus, a small amount of LCs in LC droplets close to the edges of electrode stripes cannot completely rotate to be perpendicular to the substrates [1]. Thus, the transmittance of ***x***-LPLs is reduced. Moreover, a small amount of LCs in LC droplets close to the dead zones (orange region in Figure 2b), which are the areas in the central region between two adjacent electrode stripes, cannot be oriented [1]. Accordingly, this condition also causes a reduction of transmittance of either ***x***-LPLs or ***y***-LPLs.

## 3. Preparation of PDLCs

The materials used in this study were nematic LCs, E7 (Merck), and monomers, namely, NBA107 and NOA65 (Norland Optics). Mixture A (B) was composed of E7 and NBA107 (E7 and NOA65). Table 1 lists the refractive indices of E7, cured NBA107, and cured NOA65. Table 2 shows the mechanical properties of the adopted monomers of NBA107 and NOA65. The PDLCs produced from mixtures A and B were called PDLCs_NBA107_ and PDLCs_NOA65_, respectively. The low shrinkage and low strain are the key characteristics of the adopted monomer NBA107 to minimize the potential strain [14]. Two types of empty cells were prepared. The first type of empty cell, fabricated by assembling two glass substrates coated with ITO films, was used for the experiments in Section 4.1. No alignment processes were used on the top of these two ITO-coated substrates. Empty cells with thicknesses (cell gaps) of 7 μm, defined by sprayed ball spacers, were filled with the homogeneous mixtures through capillary action. The light transmission of an empty cell was approximately 82%. Then, the PDLCs_NBA107_ and PDLCs_NOA65_ cells were produced via PIPS through UV illumination for certain optimized durations. The second type of empty cell, fabricated by assembling two glass substrates, was used for the studies in Section 4.2. One substrate was coated with patterned interdigital electrodes, whereas the other was fully coated with an ITO film. The empty cell with a thickness of 7 μm, defined by ball spacers, was filled with homogeneous mixture A with an optimized ratio by capillary action to complete the PSMLS via PIPS through UV illumination for certain optimized durations. No photo-initiator was used in all PDLC cells. Section 4.1 and Section 4.2 provide the detailed conditions of the intensity and duration of the UV illumination processes. The 365 nm central wavelength of the UV light was used to activate PIPS.

## 4. Results and Discussion

### 4.1. Investigation of the Properties of PDLCs_NBA107_

**Figure 3:**
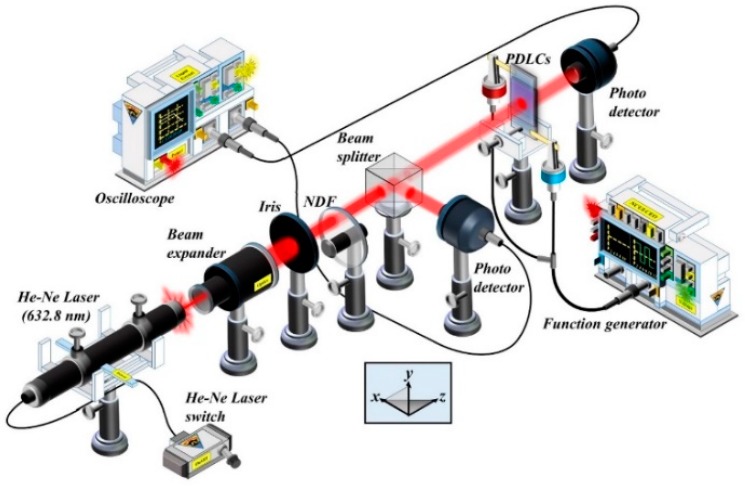
Experimental setup, drawn using Microsoft PowerPoint, for the measurement of transmission versus applied voltage (T-V) curves of PDLCs_NBA107_ and PDLCs_NOA65_.

The PDLCs_NBA107_ was studied by investigating the transmission versus applied voltage (T-V) curves of the PDLCs_NBA107_ with five different ratios of LCs (E7) and polymer (NBA107). Figure 3 shows the experimental setup for the measurement of the T-V curves. The PDLCs_NBA107_ cells (without any surface alignment treatment onto the substrates) with five different ratios of LCs to polymers were fabricated using PIPS through UV illumination with a UV intensity of 1.0 mW/cm^2^ for 60 min. The reason for selecting this particular UV intensity and illumination duration for fabrication processes will be discussed later. In Figure 3, the probe beam from a He–Ne laser was expanded by using a beam expander, and then passed through an iris. The intensity was reduced using a neutral density filter (NDF). The light was split into two beams by using a beam-splitter cube. One beam was used to measure the transmission of the PDLCs_NBA107_ cell, whereas the other beam was used to detect the intensity stability of the probed He–Ne laser. The distance between the photo-detector and the PDLC cell for all T-V curve measurements was set at around 30 cm for the baseline, and the collection angle of the scattering was about 0.8°.

**Figure 4:**
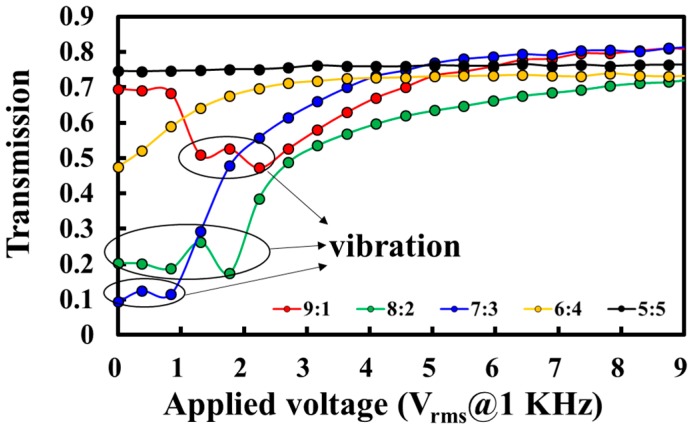
Transmission versus applied voltage (T-V) curves of PDLCs_NBA107_ with different ratios (E7:NBA107), including the weight ratios of 9:1, 8:2, 7:3, 6:4, and 5:5. The applied electric field is an alternating current (AC) square wave.

**Figure 5:**
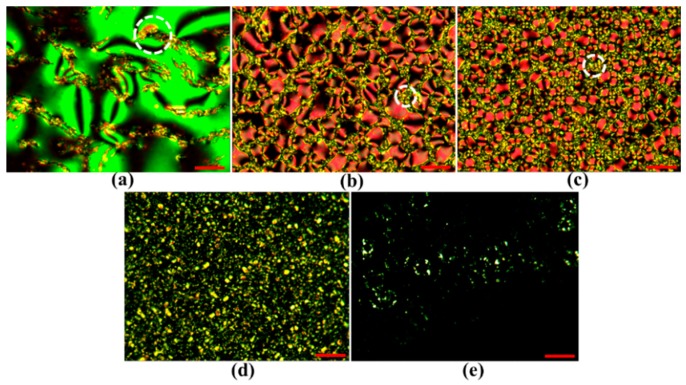
Images of PDLCs_NBA107_ with different ratios (E7:NBA107) of (**a**) 9:1, (**b**) 8:2, (**c**) 7:3, (**d**) 6:4, and (**e**) 5:5, observed under a cross-polarized optical microscope (cross-POM). The red scale bars (bottom-right side of each photo) represent a length of approximately 40 μm.

**Figure 6:**
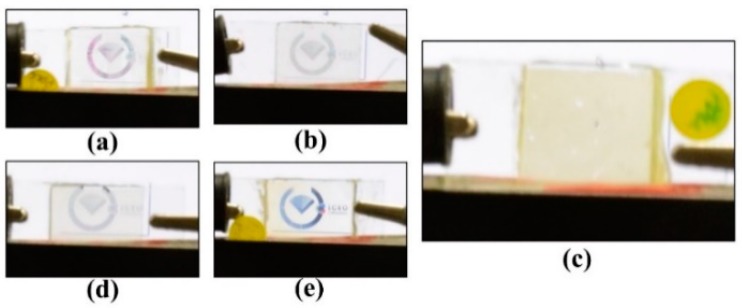
Photographs of scattering performances of different PDLCs_NBA107_ cells fabricated with different weight ratios of materials (E7:NBA107), including (**a**) 9:1, (**b**) 8:2, (**c**) 7:3, (**d**) 6:4, and (**e**) 5:5, at a zero applied-voltage state.

The red, green, blue, orange, and black curves shown in Figure 4 present the T-V curves of the PDLCs_NBA107_ with different weight ratios (E7:NBA107) of 9:1, 8:2, 7:3, 6:4, and 5:5, respectively. The experimental results of the transmissions shown in Figure 4 are based on the experimental setup shown in Figure 3. The PDLCs_NBA107_ cells were fabricated using the UV PIPS processes as described previously. Figure 5a–e present the microscopic images of the PDLCs_NBA107_ with different weight ratios (E7:NBA107) of 9:1, 8:2, 7:3, 6:4, and 5:5, respectively, observed under a cross-polarized optical microscope (cross-POM). The cell thickness of the PDLCs_NBA107_ shown in Figure 4 and Figure 5 was 7 μm. Figure 6a–e show the observations of these PDLCs_NBA107_ cells with different ratios (E7:NBA107) of 9:1, 8:2, 7:3, 6:4, and 5:5 without any applied voltage, corresponding to the initial transmissions of the red, green, blue, orange, and black curves shown in Figure 4, respectively. The detailed discussion will be provided in the next paragraph.

In Figure 5a, the concentration of the mixed monomer, NBA107, was too low to generate suitable LC droplets and a continuous polymer matrix to achieve good light-scattering for the case of [9:1]-PDLCs_NBA107_. The figure indicates that most parts of the LC cell contained LCs with various orientations. The small, polymer-rich areas (white circle in Figure 5a), which were randomly distributed, were formed through the aggregation of polymers during PIPS to cause light scattering [1]. The red curve in Figure 4 shows that the slight light loss/scattering of 13% (82% to 69%) was caused by the small polymer-rich areas and the large LC domains. For further discussion, the relatively small LC droplets in polymer-rich areas are called LC-droplets_PRA_. Some of the sizes of the LC-droplets_PRA_ were believed to be within the regime (~0.2–10 μm) to cause visible light scattering.

**Figure 7:**
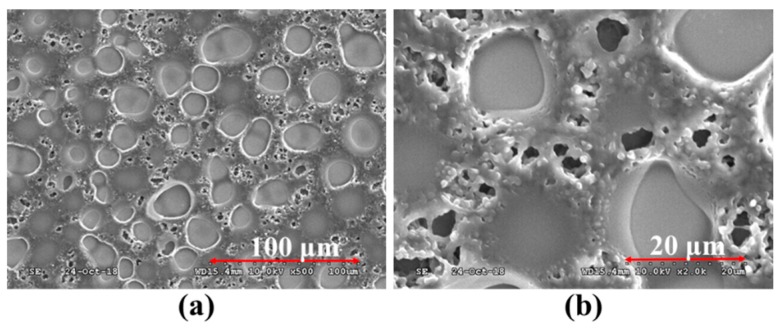
Scanning electron microscope (SEM )images of [7:3]-PDLCs_NBA107_ with magnifications of (**a**) 500× and (**b**) 2000×.

With the increase in polymer (NBA107) concentration, Figure 5b,c clearly show that the LC droplets with different sizes were generated via PIPS for the cases of [8:2]-PDLCs_NBA107_ and [7:3]-PDLCs_NBA107_, respectively. Generally, the LC droplets in these two cases were relatively much larger than the LC-droplets_PRA_ (white circles in Figure 5b,c), and thus, the large LC droplets were called LC-droplets_L_ to distinguish them from the LC-droplets_PRA_. Figure 7a,b show the scanning electron microscope (SEM) images of the [7:3]-PDLCs_NBA107_ with magnifications of 500× and 2000×, respectively. The large, circle-like area and relatively small black area indicate the size/shape of the LC-droplets_L_ and LC-droplets_PRA_, respectively. Figure 7a,b show that the size of the LC-droplets_PRA_ is approximately within 0.2–10 μm. Clearly, the size of the LC droplets shown in Figure 5b is larger than that shown in Figure 5c because the polymer concentration of the former was lower than that of the latter. However, the sizes of the LC-droplets_L_ shown in Figure 5b,c were both larger than the thicknesses of the LC cell (7 μm). This result indicates that the shapes of the LC-droplets_L_ in [8:2]-PDLCs_NBA107_ and [7:3]-PDLCs_NBA107_ were not sphere-like, but rather cylindrical. The formation of the LC-droplets_L_ grew from one substrate to the other during PIPS processes, and thus, the incident light passed through the LC-droplets_L_ areas with only slight light-scattering. Based on Figure 5 and Figure 7, the sizes of most of the LC-droplets_PRA_ of the [8:2]-PDLCs_NBA107_ and [7:3]-PDLCs_NBA107_, marked as white circles in Figure 5b,c, which are approximately in the order of 0.2–10 μm, were capable of causing visible light-scattering. Overall, as the transparent LC-droplets_L_ areas and scattering polymer-rich area in [8:2]-PDLCs_NBA107_ are larger and smaller than those in [7:3]-PDLCs_NBA107_, respectively, the initial transmission of the former (green curve in Figure 4) is higher than that of the latter (blue curve in Figure 4).

By increasing the polymer (NBA107) concentration further, we deduced that the sizes of some LC-droplets_PRA_ of the [6:4]-PDLCs_NBA107_ [Figure 5d] were not in the order of ~0.2–10 μm to cause scattering for visible lights. The polarization directions of the incident lights after passing through the non-scattering polymer-rich areas remained almost unchanged because the phase retardation contributed by the LCs in LC-droplets_PRA_ was limited and were absorbed by the analyzer of the cross-POM. Accordingly, some small black domains, shown in Figure 5d, can be observed. The sizes of some LC-droplets_L_ (light-yellow areas in Figure 5d) are larger than 7 μm, thereby allowing the incident lights to pass through the LC-droplets_L_ areas with slight light scattering. Some LC-droplets_PRA_/LC-droplets_L_ with sizes in the order of 0.2–10 μm were the main reason to cause the scattering of visible lights. Overall, the initial transmission of the [6:4]-PDLCs_NBA107_ (orange curve in Figure 4) is higher than that of the [7:3]-PDLCs_NBA107_ (blue curve in Figure 4) because some lights can pass through the [6:4]-PDLCs_NBA107_ with non-scattering polymer-rich areas and some LC-droplets_L_ areas, whose sizes are larger than 7 μm.

With an increasingly high polymer (NBA107) concentration, the sizes of most LC-droplets_PRA_ in [5:5]-PDLCs_NBA107_ were considered smaller than 0.2 μm, which did not cause scattering of visible lights. The polarization directions of the incident lights after passing through the non-scattering polymer-rich areas remained almost unchanged. Hence, Figure 5e shows the observations of an almost black state under cross-POM because most incident lights were absorbed by the analyzer. The LC-droplets_L_ (light-yellow areas in Figure 5e) with a few micrometers in size can be observed under cross-POM. However, LC-droplets with their size smaller than several hundreds of nanometers were not clearly observed because their sizes were beyond the microscope resolution limit [30]. Accordingly, the initial transmission of the [5:5]-PDLCs_NBA107_ was ~75%, which is close to the transmission (~82%) of an empty cell. The light loss of 7% was mostly caused by the light scattering resulting from the LC-droplets_L_ areas, the sizes of which were in the order of 0.2–10 μm.

Figure 4 shows that the vibrations of the transmissions of the red, green, and blue T-V curves (black circles) occurred when the applied voltages were not high enough. Based on the green and blue T-V curves in Figure 4, we infer that the LCs applied with low external fields in the center of each LC-droplets_L_/ LC-droplets_PRA_ started to rotate slightly, because the anchoring strength of the polymer surface was relatively weak there [1,2]. A small LC domain formed in the center of each LC droplet, of which the refractive index was different from the surrounding LCs, causing weak light scattering. The cause of the vibration of the red T-V curve in Figure 4 can be understood as being because the applied field induced reorientations of LCs in the central parts of large LC areas and LC-droplets_PRA_, of which the refractive indices were different from the adjacent LCs anchored by the polymer matrix to cause light scattering. Beyond the vibrations, the transmissions shown in these curves, except for the black one in Figure 4, increase with an increase of applied voltage because LCs gradually rotate to be perpendicular to the substrates through the application of various applied voltages to reduce refractive index mismatch between LCs and polymers. The increase in the transmission of the [5:5]-PDLCs_NBA107_ (black curve in Figure 4) was limited because the light-scattering source, i.e., the LC-droplets_L_ areas in Figure 5e, was extremely small. Among the curves in Figure 4, the optimized ratio of the PDLCs_NBA107_ to approach the lowest V_th_ (<1 V_rms_) and the lowest transmission (~9.4%) is 7:3.

**Figure 8:**
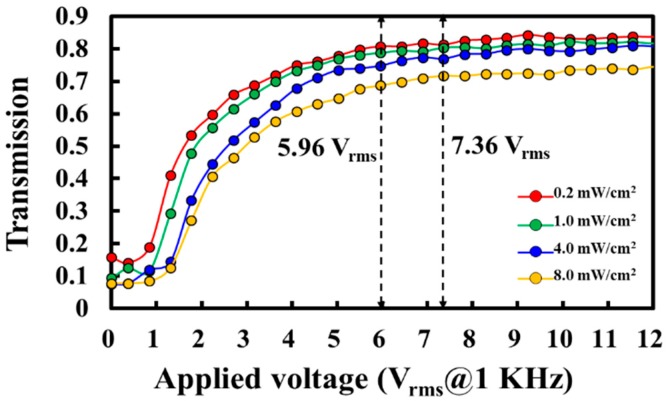
Transmission versus applied voltage (T-V) curves of different [7:3]-PDLCs_NBA107_ cells fabricated via polymerization-induced phase separation (PIPS) through the illumination of UV lights with intensities of 0.2, 1.0, 4.0, and 8.0 mW/cm^2^ for 60 min. The applied electric field is an alternating current (AC) square wave.

The selected intensity (1.0 mW/cm^2^) of UV light for PIPS in the aforementioned experiments was optimized. The red, green, blue, and yellow curves shown in Figure 8 present the T-V curves of the [7:3]-PDLCs_NBA107_ cells fabricated via PIPS through illumination of UV lights with intensities of 0.2, 1.0, 4.0, and 8.0 mW/cm^2^, respectively, for 60 min. The cell thickness of the four cells was 7 μm. In accordance with these experimental results, Table 3 lists the V_th_, initial transmission, and saturated transmission of these four [7:3]-PDLCs_NBA107_ cells. The values of V_op_, defined as the applied voltage to approach the saturated transmission, of the four [7:3]-PDLCs_NBA107_ cells shown as the red, green, blue, and orange curves in Figure 8 are considered to be approximately below 7.36 V_rms_. The saturated transmission remains almost invariant when the applied voltage is higher than V_op_.

**Figure 9:**
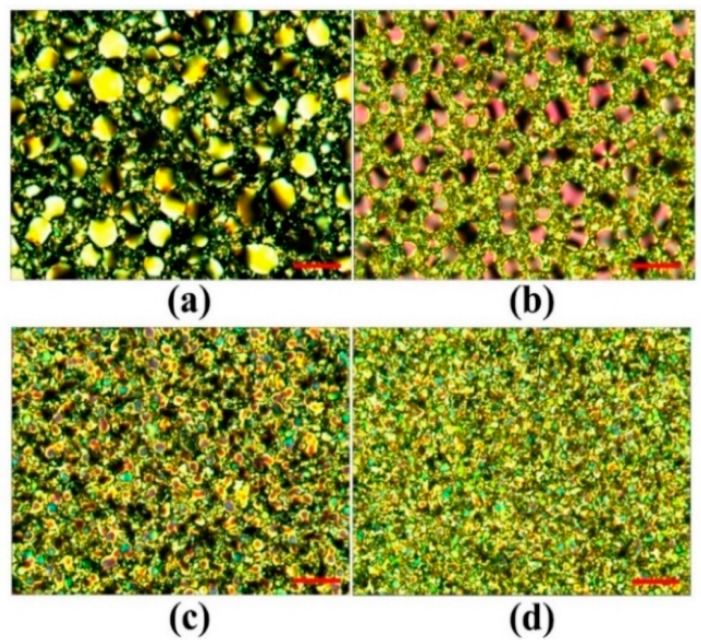
Microscopic images of the [7:3]-PDLCs_NBA107_ cells fabricated via PIPS using the illumination of UV lights with intensities of (**a**) 0.2, (**b**) 1.0, (**c**) 4.0, and (**d**) 8.0 mW/cm^2^ observed under a cross-POM. The red scale bars (bottom-right side of each photo) represent a length of approximately 40 μm.

Figure 9a–d show the microscopic images of the abovementioned [7:3]-PDLCs_NBA107_ cells fabricated via PIPS with UV intensities of 0.2, 1.0, 4.0, and 8.0 mW/cm^2^, respectively, observed under a cross-POM. In Figure 8, the initial transmission of the [7:3]-PDLCs_NBA107_ cells applied with an AC voltage of approximately 7.36 V_rms_ and the V_th_ decreased and increased with the increase of UV light intensity, respectively. Based on the images shown in Figure 9, this result is reasonable because the polymer-rich area (size of LC-droplet_L_) increased (decreased) with the increase of UV light intensity [1,2]. Moreover, a large (small) LC-droplets_L_ (polymer-rich area) suggests that less incident lights can be scattered, resulting in an increase in initial transmission.

**Figure 10:**
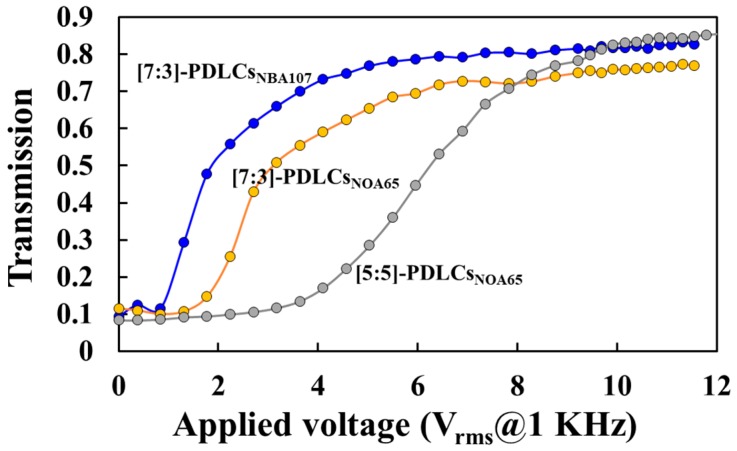
Transmission versus applied voltage (T-V) curves of the [7:3]-PDLCs_NBA107_, [7:3]-PDLCs_NOA65_ and [5:5]-PDLCs_NOA65_.

To approach low V_th_, among all curves in Figure 8, and based on Table 3, the V_th_ (<1 V_rms_) of the [7:3]-PDLCs_NBA107_ of the red and green curves were the lowest. Given that the latter had lower initial transmission than the former, the PIPS of UV illumination with the intensity of 1.0 mW/cm^2^ was selected to be the optimized fabrication condition. The V_op_ (~5.96 V_rms_) of the [7:3]-PDLCs_NBA107_ of the green curve was relatively low. Moreover, the contrast ratio of [7:3]-PDLCs_NBA107_ fabricated following the optimized processes was high enough for real applications, such as electrically switchable windows, light shutters, and e-papers [1,2]. Regarding the response time of the optimized [7:3]-PDLCs_NBA107_, its rise (decay) time was measured to be about 1.5 (193) ms. Compared with other PDLCs, the cause of such low V_th_ and relatively long decay time can be understood because the [7:3]-PDLCs_NBA107_ possesses relatively weak surface-anchoring [1,20,31,32,33]. A method to shorten the decay time in [7:3]-PDLCs_NBA107_ will be proposed in Section 4.2. Generally, PDLCs with weak surface-anchoring possess low V_th_, and the relationship between the V_th_ and LC droplet size of PDLCs with weak surface-anchoring can be described using Equation (1) [1]:

(1) $$E_{th}=\frac{V_{th}}{d}={\left(\frac{a_{surface}W}{a_{electric}\Delta \varepsilon }\right)}^{\frac{1}{2}}\frac{1}{\sqrt{D}}\propto \frac{1}{\sqrt{D}},$$

where *E_th_*, *V_th_*, *d*, *W*, *∆ε*, and *D* represent the threshold field, threshold voltage, cell thickness (gap), anchoring strength of droplet surface, LC dielectric anisotropy, and droplet size (diameter), respectively. *a_surface_* and *a_electric_* are constants. Equation (1) represents the fact that the V_th_ decreases with the increase [decrease] of UV light intensity [LC droplet size, *D*] as shown in Figure 8. To further investigate the surface anchoring conditions of the [7:3]-PDLCs_NBA107_ onto the V_th_ of PDLCs, another famous polymer of Norland Products Inc., NOA65, was selected for comparison with NBA107. We fabricated [7:3]-PDLCs_NBA107_, [7:3]-PDLCs_NOA65_ and [5:5]-PDLCs_NOA65_by using the PIPS of UV illumination (1.0 mW/cm^2^) for 60 min. The cell thickness used in the [7:3]-PDLCs_NBA107_ and [7:3]-PDLCs_NOA65_ cells was 7 μm Figure 10 illustrates the measurements of the T-V curves of the [7:3]-PDLCs_NBA107_, [7:3]-PDLCs_NOA65_ and [5:5]-PDLCs_NOA65_. Clearly, the V_th_ of the [7:3]-PDLCs_NBA107_ (blue curve) is the lowest among the three PDLCs. Moreover, the initial transmission of [7:3]-PDLCs_NBA107_ (blue curve) is slightly lower than that of the [7:3]-PDLCs_NOA65_ (orange curve). Decreasing concentration of NOA65 to increase LC droplet size can further decrease V_th_ (orange curve), but the initial transmission might increase to decrease the contrast ratio, which can refer to Figure 4 [1,2]. It is well-known that the surface anchoring of NOA65 is weak [1]. Thus, based on Figure 10, the surface anchoring of the NBA107 is believed to be weaker than or equal to that of NOA65 [1,33]. We deduce that the reason for the weak surface anchoring of the PDLCs_NBA107_ could be the low interaction force between LC and polymer molecules [20].

### 4.2. Electrically Switchable Dual-Polarization Scattering Shutter

The experimental setup for the following experiments was identical with that shown in Figure 3, except for the addition of the polarizer, which was placed between the NDF and the beam splitter. The measurement of the T-V curve of the PSMLS was achieved by rotating the transmission axis of the polarizer. The PSMLS was fabricated based on [7:3]-PDLCs_NBA107_ through PIPS with the optimized fabrication processes (UV light illumination with intensity of 1.0 mW/cm^2^ for 60 min). The scattering performances of the PSMLS when various electric potentials were applied onto the blue and light-yellow interdigital electrode stripes (Figure 2a) are discussed in the following paragraph.

**Figure 11:**
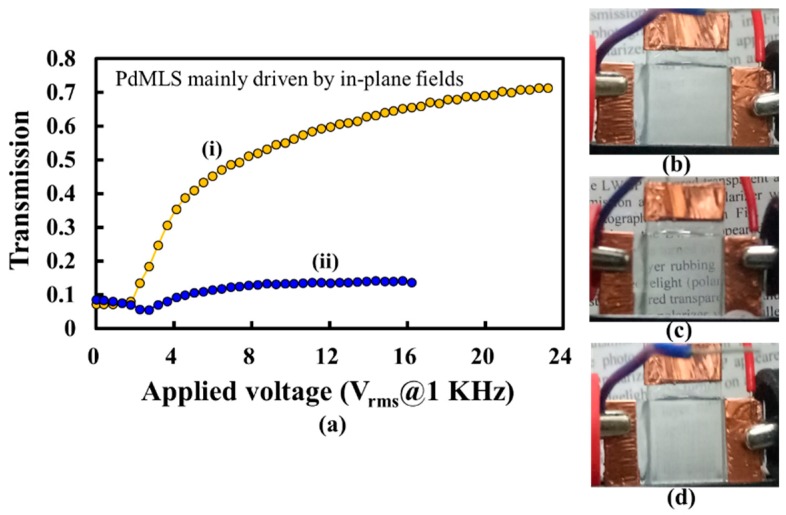
(**a**) T-V curves of PSMLS when the polarization directions of the incident lights are (i) parallel (orange) and (ii) perpendicular (blue) to the direction of the interdigital electrode stripes. (**b**) Photo of the PSMLS in the initial scattering condition (without any application of field). Photos of (**c**) transparent and (**d**) opaque PSMLS when the polarization direction of the incident lights is parallel and perpendicular to the direction of the interdigital electrode stripes (when the applied voltage is 18.5 V_rms_), respectively. The applied electric field is an AC square wave.

The orange and blue curves in Figure 11a show the T-V curves of the PSMLS when the polarization directions of the incident lights were parallel and perpendicular to the direction of the interdigital electrode stripes, respectively. The experimental results show that the lights with a polarization direction parallel to the direction of the interdigital electrode stripes can pass through the PSMLS, whereas the lights with a polarization direction perpendicular to the interdigital electrode stripes are scattered. The V_th_ of the fabricated PSMLS was lower than 2 V_rms_ because of the use of [7:3]-PDLCs_NBA107_, which was fabricated via optimized fabrication processes. Figure 11b shows the observation of the PSMLS in the initial scattering condition (without any application of an external voltage). A linear polarizer was placed between the PSMLS and camera. Figure 11c,d shows the experimental results, where the PSMLS was transparent (opaque) when the polarization direction of the incident lights was parallel (perpendicular) to the direction of the interdigital electrode stripes (when applied voltage is 18.5 V_rms_). However, based on the orange curve in Figure 11a, the V_op_ of the PSMLS was larger than 22 V_rms_, which is higher than the original V_op_ (~5.96 V_rms_) shown in Figure 8. The reason for this low original V_op_ (Figure 8) is that the applied field strength onto a common PDLC cell, assembled by using two ITO-coated glass substrates, is uniform across the bulk of the PDLC cell. This result indicates that the LCs in all LC droplets in the bulk of the cell orient at the same time through the application of an external field. By contrast, the applied field strength is reduced gradually from the bottom interdigital electrode to the top substrate in the PSMLS (Figure 2a). When specific suitable electric potentials are applied onto the blue and light-yellow interdigital electrode stripes (Figure 2a), the field strength can rotate the LCs with their director projection on the ±***x***-axis in LC droplets near the bottom interdigital electrodes (Figure 2a) to be perpendicular to the direction of the interdigital electrode stripes; however, the field strength near the top substrate is not high enough to rotate the LCs in LC droplets. Hence, the applied voltage must be increased further to rotate the LCs in all LC droplets in the bulk of PSMLS. Moreover, the blue curve (Figure 11a) shows that the transmission of the PSMLS applied with approximately 16 V_rms_ for the case of incident lights with the polarization direction perpendicular to the direction of the interdigital electrode stripes was higher than the initial transmission. This result indicates that the scattering strength is stronger when the LCs in LC droplets point to random directions than that when the LCs in LC droplets are rotated to be the configuration of Figure 2a via the applied voltage (16 V_rms_). The cause can be understood because the corresponding refractive index mismatches were different.

**Figure 12:**
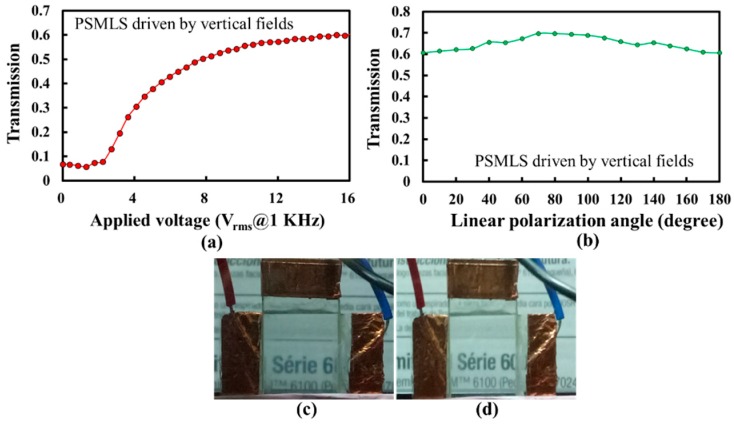
(**a**) T-V curve of the PSMLS for the incident lights with their polarization direction perpendicular to the direction of the interdigital electrode stripe. (**b**) Transmission versus linear polarization angle (LPA) curve of the PSMLS driven by vertical fields. The observations of the PSMLS applied with a vertical field of about 13.9 V_rms_ when the LPA was (**c**) 0° and (**d**) 90°. The applied electric field is an AC square wave.

The scattering performances of the PSMLS driven by the applied vertical fields, as shown in Figure 2b, are discussed in the following paragraph. Figure 12a shows the T-V curve when the polarization direction of the incident lights is perpendicular to the interdigital electrode stripes. The T-V curve in Figure 11a should be similar to that in Figure 8 because both curves were measured from the PDLC cells driven by vertical fields. However, the V_op_ and V_th_ shown in Figure 12a are higher than those shown in Figure 8 because of the dead zones with slight fringe fields (Figure 2b) in the PSMLS. The highest transmission in Figure 12a was also lower than that in Figure 8, which was elucidated in Section 2. Briefly, on the basis of Figure 2b, the fringing electric fields close to the electrode stripe edges were not completely perpendicular to the substrates, so light-scattering occurred there because of a small refractive index mismatch between LCs and polymers to reduce the transmission. A small amount of LCs in LC droplets close to the dead zones (orange region in Figure 2b), which cannot be electrically oriented, also caused the reduction of transmittance. Moreover, the V_op_ in Figure 12a is higher than that in Figure 8, which is reasonable because a high applied voltage was required to reorient LCs closer to the edges of the electrode stripes. The transmission versus linear polarization angle (LPA) curve of the PSMLS through the application of an applied voltage (~13.9 V_rms_) was measured to investigate the polarization sensitivity of the PSMLS driven by vertical fields, as shown in Figure 12b. The LPA is defined as the angle between the absorption axis of the polarizer and the direction of the interdigital electrode stripes. On the basis of Figure 2b, when the LPA is 0°/180° (90°), i.e., the incident light with the polarization direction is perpendicular (parallel) to the direction of the interdigital electrode stripe, the incident lights encounter *n*_e_/*n*_eff_ (*n*_o_) of LCs in the LC droplets close to the interdigital electrode stripe edges. As the refractive index of the polymer (NBA107) becomes closer to the *n*_o_ of nematic LC (E7), the incident lights with the polarization direction perpendicular (parallel) to interdigital electrode stripes encounter a small (~0) refractive index mismatch between polymer and LCs close to the interdigital electrode stripe edges. The incident lights with the polarization direction perpendicular to the interdigital electrode stripes should be slightly scattered. The transmission when the LPA is 0°/180° is slightly lower than that when the LPA is 90°, as shown in Figure 12b. We infer that the slightly low transmission (LPA of 0°/180°) results from the slight mismatch of refractive indices between the LCs and polymer (NBA107) close to the interdigital electrode stripe edges. Figure 12c,d show the images of the PSMLS when the LPAs are 0°/180° and 90°, respectively. A linear polarizer for the setup of Figure 12c,d was placed between the PSMLS and camera. The clarity of the words (background) in Figure 12c,d is approximately the same.

**Figure 13:**
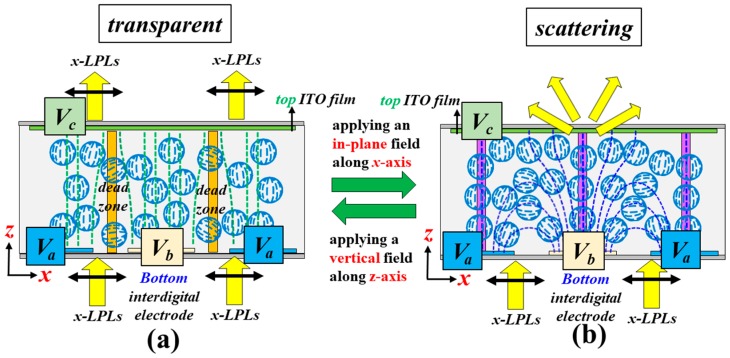
(**a**) Schematic drawings of a PSMLS when the potentials of Va, V_b_, and V_c_ are V_op_, V_op_, and 0, respectively, enabling the input ***x***-LPLs to pass through the PSMLS. (**b**) Schematic drawings of a PSMLS when the potentials of Va, V_b_, and V_c_ are 0, V_op_, and open circuit potential (floating), respectively, to obtain the light scattering of ***x***-LPLs.

The [7:3]-PDLCs_NBA107_ can realize low V_th_ and V_op_ due to its weak surface-anchoring, but the cost is a relatively long decay time (~193 ms). To apply [7:3]-PDLCs_NBA107_ for PSMLSs, a method to shorten its decay time is elucidated as follows. Based on the results in Figure 11c,d, we proposed the method to approach a fast switch between a polarization-selective scattering state and polarization-independent transparent state. Figure 13a shows that when the PSMLS is applied with a vertical field, the input ***x***-LPLs can pass through the PSMLS (Figure 12c). The potentials of V_a_, V_b_, and V_c_ to obtain the transparent state, as shown in Figure 13a, are V_op_, V_op_, and 0, respectively. Figure 13b shows that to obtain the light scattering of ***x***-LPLs (Figure 11d), the potentials of V_a_, V_b_, and V_c_ of the PSMLS are 0, V_op_, and open circuit potential (floating), respectively. Because the switch from transparent to scattering states of ***x***-LPLs in PSMLS is driven by the applied electric fields rather than the weak surface anchoring in each LC droplet, the switch time can be further shortened [1,22,23]. By contrast, the required time of switch from transparent to scattering states in a common PDLC device depends only on surface anchoring in each LC droplet; therefore, its switch time is relatively long [1,2]. To simplify the design to approach this method, the interdigital electrode stripes can be replaced by using a configuration consisting of grid electrodes located on top of a common electrode [22,23].

## 5. Conclusions

Low-threshold-voltage, low-operation-voltage, and electrically switchable PSMLSs using [7:3]-PDLCs_NBA107_ were demonstrated in this work. In addition to the optimized conditions for fabrication processes, the electro-optical properties, including scattering performance, V_th_, initial transmission, surface anchoring, and droplet size, of the [7:3]-PDLCs_NBA107_ were investigated. The full and systematical analysis of [7:3]-PDLCs_NBA107_ can be a useful reference for further PDLCs studies. The relationship between the V_th_ and LC droplet size of PDLCs based on surface anchoring was discussed. The V_th_ and V_op_ of the 7 μm-thick [7:3]-PDLCs_NBA107_ cells produced via optimized fabrication processes are lower than 1 and 6 V_rms_, respectively. Moreover, the contrast ratio of the [7:3]-PDLCs_NBA107_ is ~9. Accordingly, the [7:3]-PDLCs_NBA107_ with low power consumption has considerable potential to be applied in the various areas of optics [1,2,3,4,5,6,7,8,9,10,11,12]. The scattering performances of the PSMLS mainly driven by in-plane and/or vertical fields with low V_th_ were also demonstrated. The PSMLS mainly driven by the in-plane field can scatter lights with the polarization direction perpendicular to the direction of the interdigital electrode stripes, whereas the scattering of the PSMLS driven by the vertical field is insensitive to the polarization direction of incident lights. Accordingly, the PSMLS can be adopted to be an electrically switchable PSMLS for linearly polarized or unpolarized lights for projection or 3D display applications. The weak surface anchoring of [7:3]-PDLCs_NBA107_ can realize low V_th_ and V_op_, but the cost is a long decay time. When [7:3]-PDLCs_NBA107_ is applied for PSMLSs, to reduce decay time, the switch from transparent to scattering states in PSMLS can be driven by the applied electric fields rather than the weak surface anchoring of the adopted polymer. Hence, the fast switch between a polarization-selective scattering state (Figure 11d) and polarization-independent transparent state (Figure 12c) in the PSMLS is feasible with a suitable electrode design [22,23,24,25]. Also, the response time of a switch between polarization-independent light scattering and transparency based on PDLCs can be reduced according to the similar method, with two substrates having orthogonally interdigital electrode stripes [34].

## Figures and Tables

**Table 1:** Refractive indices of E7, NBA107, and NOA65.

E7	NOA65	NBA107
Extraordinary (ordinary) refractive index	Refractive index after polymerization	Refractive index after polymerization
1.737 (1.5185) [20 °C, λ = 633 nm]	1.524	1.51

**Table 2:** Mechanical properties of NBA107 and NOA65.

	Viscosity (25°)	Modulus of Elasticity (psi)	Tensile Strength (psi)	Elongation at Failure	Hardness-Shore D
NBA107	350 cps	800	78	5%	15
NOA65	1200 cps	20,000	1500	80%	50

**Table 3:** Threshold voltage (V_th_), initial transmission, and saturated transmission of different [7:3]-PDLCs_NBA107_ cells fabricated via PIPS using the illumination of UV lights with four different intensities.

UV Light Intensity (mW/cm^2^)	V_th_ (V_rms_)	Initial Transmission	Saturated Transmission
0.2	<1.0	0.158	0.812
1.0	<1.0	0.094	0.804
4.0	<1.5	0.075	0.769
8.0	<1.5	0.077	0.717
